# MiR‐146a engineered extracellular vesicles derived from mesenchymal stromal cells more potently attenuate ischaemia–reperfusion injury in lung transplantation

**DOI:** 10.1002/ctm2.70298

**Published:** 2025-04-07

**Authors:** Xiucheng Yang, Shanchao Hong, Tao Yan, Mingzhao Liu, Mingyao Liu, Jin Zhao, Bingqing Yue, Di Wu, Jingbo Shao, Man Huang, Jingyu Chen

**Affiliations:** ^1^ Lung Transplantation Center Second Affiliated Hospital Zhejiang University School of Medicine Hangzhou Zhejiang China; ^2^ Department of Clinical Laboratory Jiangnan University Medical Center Wuxi China; ^3^ Wuxi Lung Transplant Center Wuxi People's Hospital Affiliated to Nanjing Medical University Wuxi Jiangsu China; ^4^ Department of Thoracic Surgery People's Hospital of Rizhao Rizhao China; ^5^ Toronto General Hospital, Research Institute University Health Network Toronto Ontario Canada; ^6^ Department of General Intensive Care Unit the Second Affiliated Hospital of Zhejiang University School of Medicine Hangzhou Zhejiang China; ^7^ Key Laboratory of Early Warning and Intervention of Multiple Organ Failure Ministry of Education of the People's Republic of China Hangzhou Zhejiang China

**Keywords:** extracellular vesicles, ischaemia–reperfusion injury, lung transplantation, miR‐146a

## Abstract

**Background:**

The limited donor lung pool for lung transplantation (LTx) is largely due to concerns over ischaemia–reperfusion injury (IRI), a major cause of primary graft dysfunction (PGD). NLRP3 inflammasome activation is known to play a pivotal role in the onset of IRI. While human umbilical cord mesenchymal stromal cell‐derived extracellular vesicles (hucMSC‐EVs) have shown potential in reducing acute lung injury, their effects on NLRP3 activation in the context of LTx remain unclear.

**Methods:**

In this study, engineered hucMSC‐EVs were delivered via nebulisation to mitigate IRI in rat LTx models. We utilised both a rat orthotopic LTx model and a cell cold preservation reperfusion model to evaluate the therapeutic efficacy of hucMSC‐EVs. Bulk‐RNA sequencing, single‐cell sequencing analysis, immunofluorescence and Western blot techniques were employed to assess NLRP3 inflammasome activation and inflammation.

**Results:**

Nebulised hucMSC‐EVs were efficiently internalised by alveolar macrophages (AMs), significantly reducing lung injury and improving oxygenation in the LTx models. Mechanistically, the engineered hucMSC‐EVs, which enhance the expression of miR‐146a, can more effectively suppress the activation of the NLRP3 inflammasome by targeting the IRAK1/TRAF6/NF‐κB pathway, resulting in decreased levels of IL‐1β, IL‐18 and other inflammatory cytokines. These findings highlight the potential of miR‐146a‐modified EVs in modulating innate immune responses to alleviate IRI.

**Conclusion:**

Our results demonstrate that nebulised delivery of engineered hucMSC‐EVs effectively mitigates IRI in LTx by inhibiting NLRP3 inflammasome activation. This innovative approach presents a promising strategy for enhancing donor lung preservation and improving post‐transplant outcomes in LTx.

**Highlights:**

- **Nebulized Delivery of miR‐146a Engineered hucMSC‐EVs Mitigates Ischemia‐Reperfusion Injury (IRI) in Lung Transplantation**. This study demonstrates the therapeutic potential of nebulized, engineered human umbilical cord mesenchymal stromal cell‐derived extracellular vesicles (hucMSC‐EVs) modified with miR‐146a to alleviate IRI in rat lung transplantation models. The treatment significantly improved lung oxygenation and reduced inflammation, highlighting the efficacy of this novel approach in enhancing donor lung preservation.
- **Mechanistic Insights: Inhibition of NLRP3 Inflammasome Activation**. Engineered hucMSC‐EVs efficiently targeted alveolar macrophages and suppressed NLRP3 inflammasome activation through the IRAK1/TRAF6/NF‐κB pathway. This modulation of innate immune responses played a crucial role in reducing IRI‐induced lung injury and inflammation, offering a promising strategy to manage primary graft dysfunction in lung transplantation.
- **Superior Efficacy of miR‐146a‐Modified EVs in Reducing Inflammatory Cytokines**. The miR‐146a modification enhanced the anti‐inflammatory properties of hucMSC‐EVs, leading to a more significant reduction in pro‐inflammatory cytokines (IL‐1β, IL‐18, and TNF‐α) compared to unmodified EVs. This targeted intervention presents a potential therapeutic avenue for improving lung transplant outcomes and mitigating IRI.
- **Innovative Therapeutic Approach: Non‐Invasive Nebulization for Direct Lung Delivery**. The use of nebulized EVs for direct delivery to donor lungs represents a non‐invasive and efficient method for lung‐targeted therapy. This strategy could expand the applicability of MSC‐EV‐based treatments for improving lung transplantation outcomes, particularly in enhancing donor lung preservation during the procurement process.

## Graphical abstract

Nebulised miR‐146a‐engineered hucMSC‐EVs reduce lung IRI in transplantation by inhibiting NLRP3 inflammasome activation through the IRAK1/TRAF6/NF‐κB pathway, improving graft function and reducing inflammation.

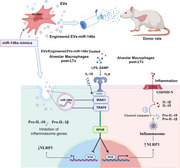

## 1 INTRODUCTION

Donor lung scarcity remains a significant challenge for lung transplantation (LTx) globally,^1^ with utilisation rates below 20% largely due to the risk of primary graft dysfunction (PGD). PGD is a major contributor to early postoperative mortality and effective treatments are limited. Enhancing donor lung preservation is essential for expanding the donor pool and improving transplant outcomes. While post‐transplant interventions are important, existing studies indicate that optimised pre‐transplant lung management can reduce PGD incidence and support successful LTx. Therefore, advancing preservation strategies is crucial to address organ shortages and enhance the effectiveness of LTx.^2^, ^3^

Lung ischaemia–reperfusion injury (IRI) is a major trigger of PGD, initiating an inflammatory innate immune response.^4^, ^5^, ^6^ NLRP3 inflammasome activation in alveolar macrophages (AMs) has been identified as a key driver of acute lung injury.^7^, ^8^, ^9^ Gene expression analyses in lung transplant patients further implicate innate immune pathways (NLRs and TLRs) in the development of PGD.^10^ However, the specific contribution of AM NLRP3 activation to lung IRI after LTx remains incompletely understood.

Mesenchymal stromal cells (MSCs) are a type of multipotent cell capable of repairing damaged tissues, predominantly through the secretion of paracrine factors.^11^ As paracrine mediators, extracellular vesicles (EVs) mitigate inflammatory responses and prevent cell death. MSC‐derived EVs (MSC‐EVs) with diameters ranging from 30–150 nm hold great promise for cell‐free therapies owing to their anti‐inflammatory and reparative properties.^12^ As crucial mediators of information exchange between cells, EVs mediate functions through their packaged parental cell proteins, miRNAs and lipids. miRNAs are a type of small non‐coding RNA that are key regulators of RNA silencing through post‐transcriptional regulation. In one study, human umbilical cord‐derived MSCs (huc‐MSCs) were able to inhibit nucleus pulposus cell pyroptosis through METTL14/NLRP3 via the transfer of exogenous miR‐26a‐5p.^13^ A different study found that MSC‐EVs reduce lung IRI and improve donor lung reconditioning after circulatory death.^14^

However, natural EVs often lack specific targeting capabilities and consistent therapeutic efficacy. To overcome these limitations, genetic engineering and modification techniques are being increasingly employed. Sequencing of huc‐MSCs revealed high expression of miR‐146a, which has been recognised as a classical therapeutic target for age‐related diseases, including tendinopathy and Alzheimer's disease.^15^, ^16^ While miR‐146a simultaneously exerting protective effects in sepsis‐induced acute lung injury and renal IRI,^17^ acting as a negative regulator of innate immune signalling by targeting key upstream mediators of the NF‐κB pathway (e.g., IRAK1 and TRAF6).^18^ Based on this, we encapsulated miR‐146a into hucMSCs‐derived EVs (EVs‐miR‐146a) to improve therapeutic effectiveness. Nebulised inhalation of MSC‐EVs is a new targeted delivery method, especially in LTx. It enables direct delivery of therapeutic substances to donor lungs, representing an innovative application in MCS‐EVs‐mediated therapies. Furthermore, capitalising on the strong phagocytic activity of AMs, we adopted a macrophage‐targeting strategy through nebulised inhalation of EVs‐miR‐146a to directly deliver therapeutic agents to donor lungs.

Using a rat model of orthotopic left LTx, compared with huc‐MSCs‐derived EVs, EVs‐miR‐146a achieved better therapeutic outcomes, alleviating lung injury and the inflammatory response. Mechanistically, overexpressing miR‐146a in EVs more significantly inhibited the IRAK1/TRAF6/NF‐κB pathway, thus reducing NLRP3 inflammasome activation. Overall, our novel therapeutic system combining engineered exosome‐based interventions and aerosol delivery technology holds promise for alleviating donor lung IRI.

## 2 RESULTS

### 2.1 Isolated hucMSC‐EVs mitigate IRI in a rat LTx model

**FIGURE 1:**
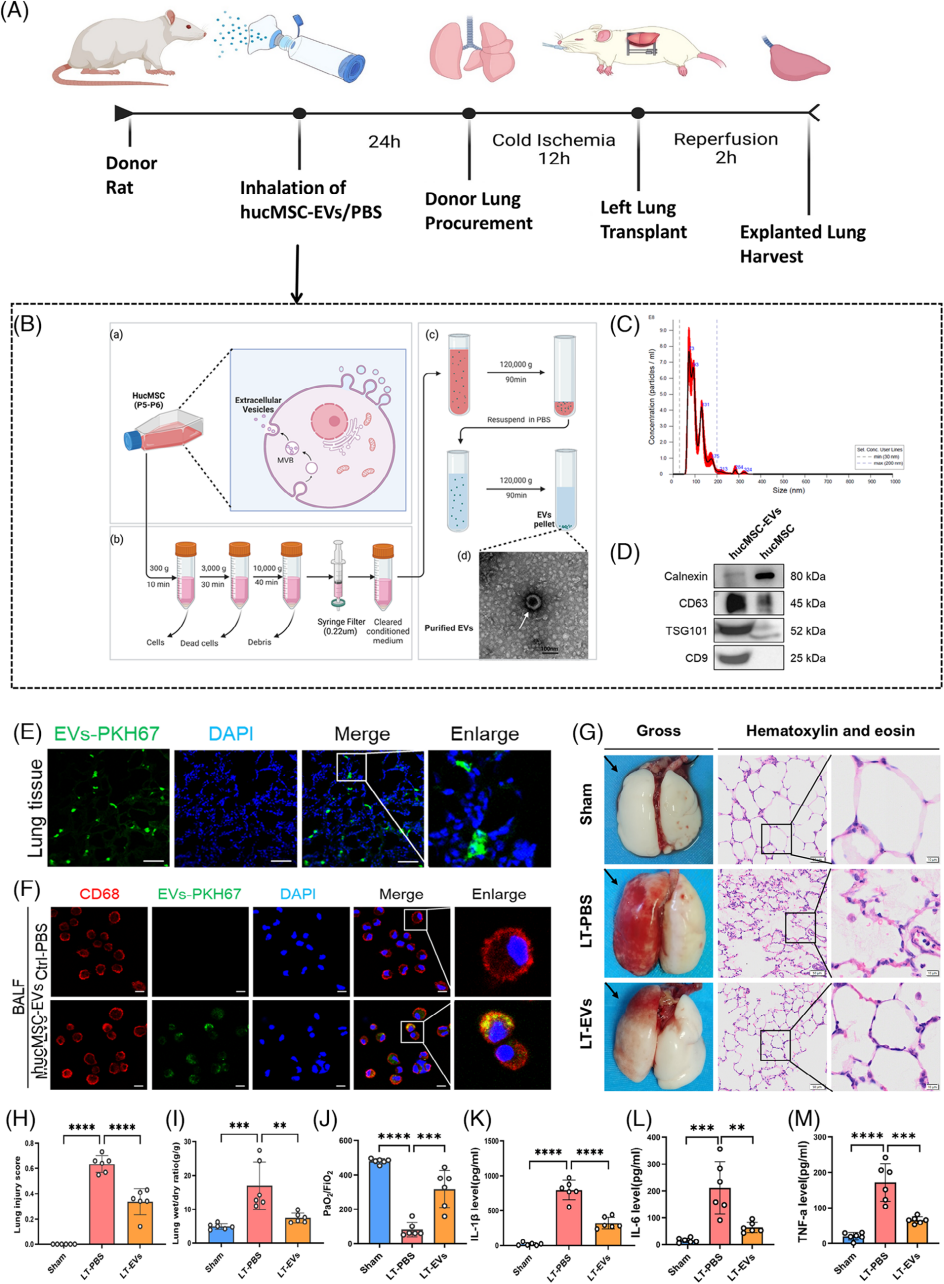
Isolated hucMSC‐EVs mitigate ischemia–reperfusion injury in a rat lung transplantation model. (A) Flowchart summarising the intervention procedure for donor rats with aerosolised hucMSC‐EVs or PBS. Donor lungs were perfused with low‐potassium dextran (LPD) solution 24 h post‐intervention, harvested and stored at 4°C for 12 h. Orthotopic left lung transplantation was performed, and the transplanted lung was harvested 2 h after reperfusion and flushed with saline via the pulmonary artery. (B) (a–c) Workflow illustrating the isolation of hucMSC‐EVs by differential ultracentrifugation. (d) Transmission electron microscopy (TEM) image showing the characteristic cup‐shaped morphology of EVs (scale bar = 100 nm). (C) Particle size distribution of hucMSC‐EVs, measured using nanoparticle tracking analysis (NTA), showing the average size of the EVs. (D) Western blot analysis of hucMSC‐EVs showing the presence of positive markers (TSG101, CD9, CD63) and the absence of the negative marker (calnexin). (E) PKH67‐labelled hucMSC‐EVs (green) administered via aerosol inhalation were widely distributed in the lung tissue of donor rats, as shown by immunofluorescence (scale bar = 50 µm). (F) Uptake of typical images show PKH67‐labelled hucMSC‐EVs (green) localised in CD68‐positive alveolar macrophages (AM) in bronchoalveolar lavage fluid (BALF) of explanted lungs (scale bar = 10 µm). (G) Gross examination and H&E staining images of the left lung following LTx, with arrows indicating the left explanted lung (scale bar = 50 µm; inset, magnification, scale bar = 10 µm). (H) Quantification of lung injury scores based on H&E staining to evaluate the severity of lung pathology. (I) Analysis of the wet/dry ratio of lung tissue. (J) Measurement of the PaO_2_/FiO_2_ from blood collected from the left pulmonary vein. Compared with the PBS‐treated group, hucMSC‐EVs treatment significantly reduced lung injury scores and wet/dry ratios, while significantly increasing the pulmonary venous PaO_2_/FiO_2_ (*n* = 6). (K–M) Levels of inflammatory cytokines IL‐1β, IL‐6 and TNF‐α, as measured by ELISA. The expression of these cytokines was significantly reduced in the hucMSC‐EVs‐treated group compared with the LT‐PBS group (*n* = 6). Sham, sham surgical group of rats; LT‐PBS, lung transplantation group of rats tread with PBS; LT‐EVs, lung transplantation group of rats tread with hucMSC‐EVs. All data are presented as mean ± SD. *p < .05, **p < .01, ***p < .001, ****p < .0001. Statistical comparisons between groups were performed using unpaired Student's *t*‐tests.

An in situ left LTx model was established. To determine whether nebulising hucMSC‐EVs in donor rats could protect the lungs from IRI during LTx, we adopted a treatment protocol as depicted in Figure 1A. The actual operation images of rat nebulisation are provided in Figure S1. MSCs were isolated from the Wharton's jelly of human umbilical cords using the tissue explant adherence method. We confirmed the successful acquisition of huc‐MSCs by examining cell characteristics, including their spindle‐shaped morphology, MSC‐specific surface markers and multipotent differentiation potential (Figure S2). The hucMSC‐EVs were subsequently isolated from huc‐MSCs through differential and ultra‐high speed centrifugation (Figure 1B, a–c). TEM revealed that hucMSC‐EVs displayed the characteristic cup‐shaped morphology typical of EVs (Figure 1B, d). Nanoparticle tracking analysis (NTA) indicated an average concentration of 4.14 × 10^10^ ± 1.60 × 10^9^ particles per millilitre (Figure 1C). Western blotting verified exosomal markers CD9, CD63 and TSG101, while the absence of the organelle‐specific marker calnexin further affirmed the purity of the isolated hucMSC‐EVs (Figure 1D).

Twenty‐four hours after nebulised inhalation of PKH67‐labelled hucMSC‐EVs, immunofluorescence staining revealed a widespread distribution of PKH67‐labelled EVs in lung tissues (Figure 1E). In addition, immunofluorescence staining revealed that PKH67‐labelled EVs were localised in CD68‐positive AMs within the BALF. The positive endocytosis rate within 24 h was 94.1% (Figure 1F). These results indicate that AMs in lung tissues can efficiently take up hucMSC‐EVs. Subsequently, orthotopic left LTx was carried out in rats. After 2 h of reperfusion, the explanted lungs were harvested. Gross inspection showed that the left lung grafts treated with PBS exhibited severe pulmonary congestion and oedema. In contrast, these pathological manifestations were significantly alleviated in the group treated with hucMSC‐EVs; H&E staining demonstrated diffuse alveolar damage, which was characterised by the disruption of alveolar walls, alveolar oedema, as well as the infiltration of monocytes/macrophages and red blood cells. Significantly, these alterations were remarkably lessened in the hucMSC‐EVs‐treated group (Figure 1G).

Quantitative analysis revealed that the hucMSC‐EVs treated group exhibited significantly lower lung injury scores and wet/dry ratios compared with the PBS‐treated group (Figure 1H,I). The photo of lung oedema in rats after LTx are provided in Figure S3. To assess the functionality of the explanted lung, the PaO_2_/FiO_2_ of blood in the left pulmonary vein was measured. The findings demonstrated that the hucMSC‐EVs treated group demonstrated a significantly increased PaO_2_/FiO_2_ than the control group (Figure 1J). Additionally, lung tissue inflammatory cytokine levels (IL‐1β, IL‐6 and TNF‐α) were markedly higher in the PBS group than in the hucMSC‐EVs group (Figures 1K–M).

### 2.2 hucMSC‐EVs alleviate IRI in rat LTx model possibly by mitigating NLRP3 inflammasome activation in AMs

**FIGURE 2:**
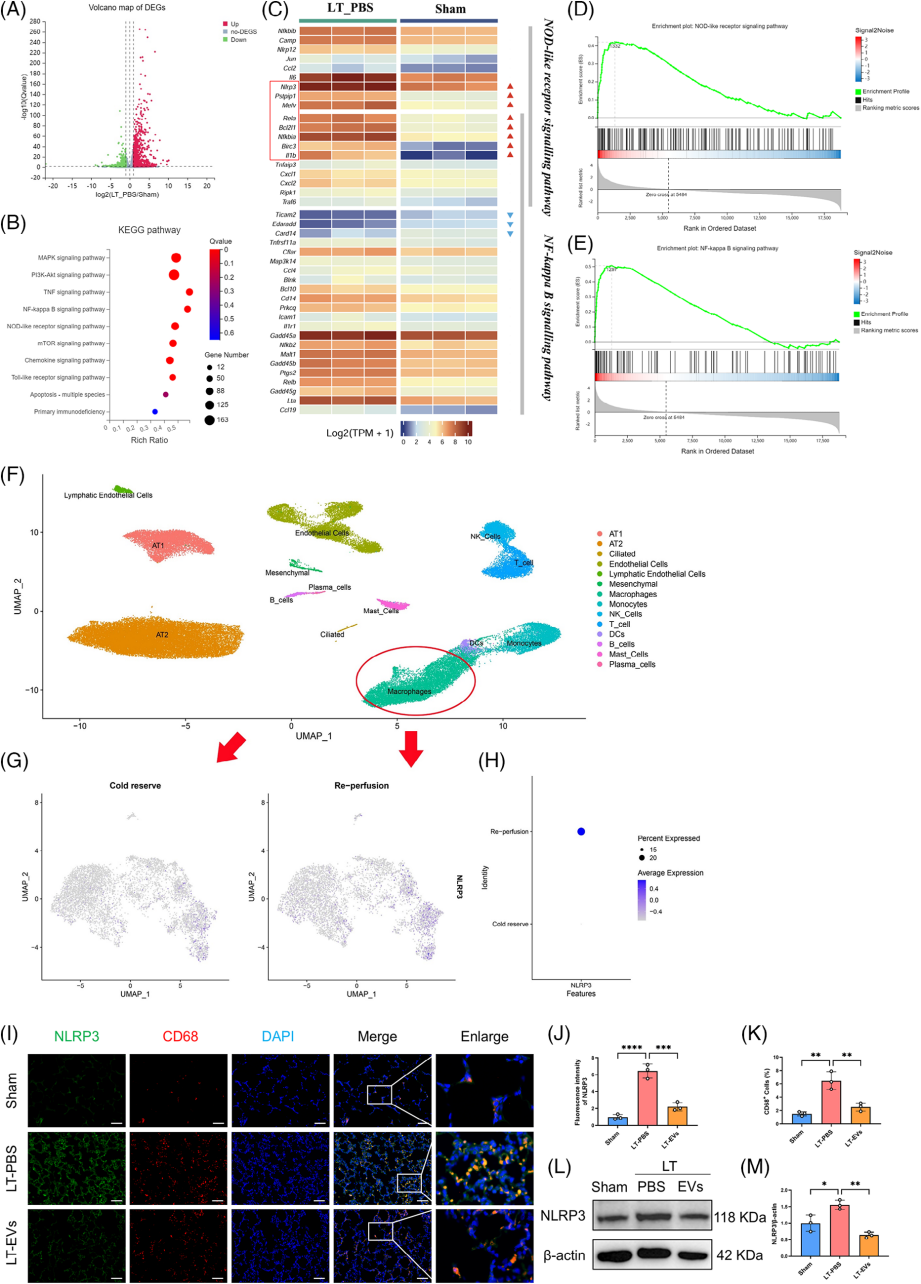
hucMSC‐EVs alleviate IRI in rat lung transplantation model possibly by mitigating NLRP3 inflammasome activation in AMs. (A) A volcano plot showing the DEGs (fold change > 1; *q*‐value < 0.05;) in the LT and sham groups (*n* = 3). (B) KEGG analysis of DEGs for 10 of the pathways with significant enrichment are shown. (C) Heat map of NOD‐like receptor signalling pathway and NF‐κB signalling pathway‐related genes. (D) GSEA analysis for NOD‐like receptor signalling pathway. (E) GSEA analysis for NF‐κB signalling pathway. (F) The Umap exhibited human scRNA‐seq atlas for pulmonary ischemia–reperfusion injury in lung transplantation. (G and H) FeturePlot and DotPlot showed the difference of NLRP3 in macrophages between cold reserve and reperfusion group. (I) Representative images displaying double immunostaining with CD68 (macrophages marker, red) and NLRP3 (green) (scale bar = 50 µm). (J) Fluorescence intensity of NLRP3 in each group. (K) Quantification of the proportion of CD68‐positive cells. (L and M) Western blot images depicting the protein levels of NLRP3 in the sham, LT‐PBS and LT‐EVs groups, along with the quantification of Western blot bands. Sham, sham surgical group of rats; LT‐PBS, lung transplantation group of rat tread with PBS; LT‐EVs, lung transplantation group of rat tread with hucMSC‐EVs. All data are presented as the mean ± SD. *p < .05, **p < .01, ***p < .001, ****p < .0001, compared with the LT‐PBS treatment group by unpaired Student's *t*‐tests.

To explore the possible mechanism of IRI in LTx, lung tissues from grafts and a sham group were collected for RNA‐Seq analysis. A volcano plot was used to display the differentially expressed genes (DEGs) between the two groups (Figure 2A). Compared with the sham group, the LT‐PBS group had 3520 up‐regulated and 2715 down‐regulated transcripts. KEGG analysis identified 10 signalling pathways, such as the NF‐κB and NOD‐like receptor signalling pathways (Figure 2B). Thirty key genes from these two pathways were selected to create an expression cluster heatmap (Figure 2C). It showed that many genes in the NOD‐like receptor and NF‐κB signalling pathways were differentially expressed in LTx rat model compared with the sham group. Gene enrichment analysis (GSEA) was performed on NOD‐like receptor and NF‐κB signalling pathway, respectively, which are involved in NLRP3 inflammasome formation and activation (Figure 2D,E).

To validate the findings from the rat experiment described above, we generated a human single‐cell RNA sequencing (scRNA‐seq) atlas of IRI in LTx^19^ (Figure 2F). Focusing on macrophage subtypes, we found that both the positive ratio and expression level of NLRP3 were markedly elevated in the reperfusion group (Figure 2G,H). Our findings indicate that macrophage‐expressed NLRP3 may be a key mediator in donor lung IRI. Thus, we found NLRP3 expression was mainly concentrated in AMs (CD68 positive, CD11b negative) within lung tissue using immunofluorescence staining (Figure S4). Meanwhile, the majority of NLRP3 was localised in CD68‐positive macrophages, with significantly increased levels of expression observed in the PBS‐treated group compared with the hucMSC‐EVs‐treated group (Figures 2I–K). Additionally, Western blot analysis confirmed a significant increase in NLRP3 protein levels in the PBS‐treated group relative to the hucMSC‐EVs‐treated group (Figure 2L,M).

### 2.3 Select miR‐146a as overexpression target to construct engineered hucMSC‐EVs

**FIGURE 3:**
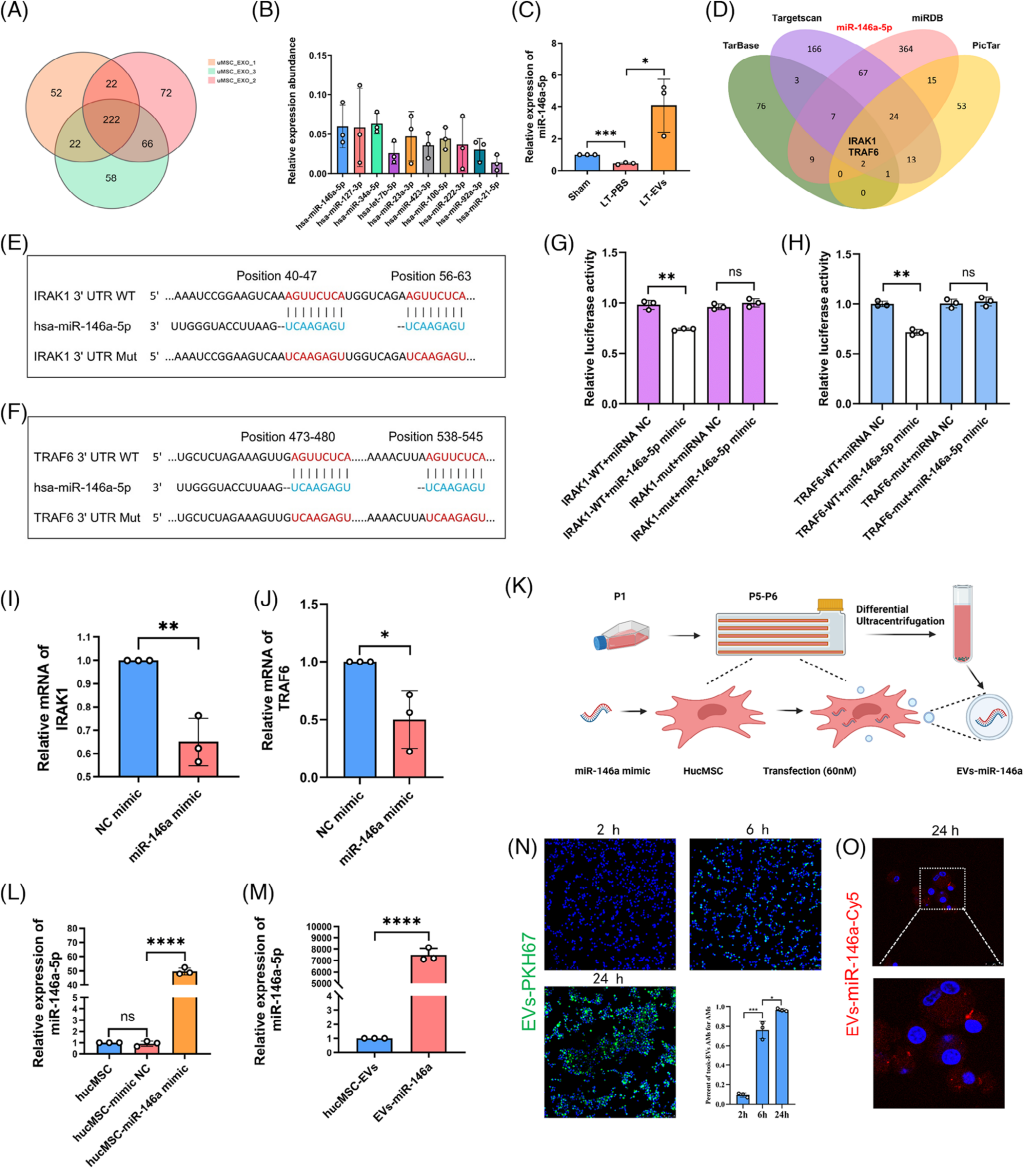
Characterise miRNA profiles in hucMSC‐EVs and construct engineered hucMSC‐EVs with miR‐146a overexpression. (A) Venn diagram illustrating the overlap of small RNA sequencing results from three hucMSC‐EVs samples, showing 222 commonly expressed small RNAs. (B) Top 10 most abundant miRNAs in hucMSC‐EVs, highlighting the high expression of miR‐146a‐5p, known for its anti‐inflammatory properties. (C) Relative expression levels of miR‐146a‐5p in PBS‐treated and EVs‐treated rat LTx models. (D) Venn diagram depicting the overlap among four miRNA prediction databases (TarBase, TargetScan, miRDB and PicTar), identifying miR‐146a‐5p as a target for both IRAK1 and TRAF6. (E and F) Schematic representation of miR‐146a‐5p binding to the wild‐type or mutated 3′‐UTR regions of IRAK1 and TRAF6. (G and H) Dual‐luciferase reporter assays validating the binding of miR‐146a‐5p to the 3′‐UTR of IRAK1 and TRAF6 mRNAs in HEK‐293T cells. (I and J) Reduced expression of IRAK1 and TRAF6 in NR8383 cells transfected with 50 nM miR‐146a mimics (double‐stranded miR‐146a duplex). (K) Flowchart showing the transfection of hucMSCs with 60 nM miR‐146a mimics, leading to the construction of engineered EVs‐miR‐146a. (L) Relative expression levels of miR‐146a‐5p in hucMSCs transfected with 60 nM miR‐146a mimics. (M) Relative expression levels of miR‐146a‐5p in engineered EVs‐miR‐146a compared with hucMSC‐EVs. (N) Fluorescence microscopy showing time‐dependent uptake of PKH67‐labelled hucMSC‐EVs (green) by NR8383 cells at 2, 6 and 24 h (scale bar = 100 µm). (O) Immunofluorescence images showing NR8383 cells incubated with hucMSC‐EVs‐cy5‐miR‐146a (red) for 24 h. Sham, sham surgical group of rats; LT‐PBS, lung transplantation group of rat tread with PBS; LT‐EVs, lung transplantation group of rat tread with hucMSC‐EVs. All data are presented as mean ± SD. *p < .05, **p < .01, ***p < .001, ****p < .0001. Statistical comparisons between groups were performed using unpaired Student's *t*‐tests.

To elucidate the impact of hucMSC‐EVs on IRI in LTx, we performed miRNA sequencing on hucMSC‐EVs from three independent biological replicates. A total of 222 miRNAs were identified across the three preparations (Figure 3A), with the ten most abundant miRNAs highlighted in Figure 3B. Among these, miR‐34a‐5p, miR‐146a‐5p and miR‐127‐3p rank as the top three miRNAs with high expression abundance. Previously, research on miR‐34a‐5p mainly focused on tumourigenesis and development. For example, miR‐34a‐5p acts as a tumour suppressor in cutaneous squamous cell carcinoma cells by targeting SIRT6.^20^ Regarding miR‐146a‐5p, it functions as a critical modulator of inflammatory and immune responses. For example, miR‐146a‐5p suppresses the TLR4/NF‐κB pathway, thereby mitigating intestinal inflammation. Moreover, miR‐146a‐5p can also regulate intestinal homeostasis.^21^ Our findings confirmed that following LTx, the LT‐PBS group exhibited a reduced expression of miR‐146a‐5p compared with the sham group. However, after pre‐treatment with nebulised hucMSC‐EVs, the expression level of miR‐146a‐5p increased in the LT‐EVs group (Figure 3C). The experimental findings indicate that miR‐146a‐5p plays a critical role in the hucMSC‐EVs‐mediated amelioration of donor lung IRI during LTx.

Bioinformatic analysis indicated that IRAK1 and TRAF6 were identified as putative targets of miR‐146a‐5p (Figures 3D–F). This was validated through luciferase reporter assays (Figure 3G,H), and NR8383 cells transfected with a miR‐146a mimic displayed a marked reduction in IRAK1 and TRAF6 mRNA levels, as determined by RT‐qPCR (Figure 3I,J).

However, exogenous miR‐146a‐5p can induce a robust immune response via TLR7 activation through its UU‐containing motif. Therefore, directly introducing miR‐146a‐5p mimic**s** into hucMSC‐EVs may not be optimal. Instead, we selected double‐stranded miR‐146a as the target miRNA for constructing engineered EVs, as its precursor does not activate the immune response. Next, we constructed engineered EVs by transfecting hucMSCs with a miR‐146a mimic (Figure 3K). Twenty‐four hours after transfection, hucMSCs transfected with miR‐146a‐5p exhibited a significant up‐regulation in its expression compared with both non‐transfected cells and those transfected with a negative control mimic, as determined by RT‐qPCR (Figure 3L). After 48 h, the cell culture supernatant from exosome‐free foetal bovine serum (FBS) medium was harvested, and engineered hucMSC‐EVs (EVs‐miR‐146a) isolated via differential ultracentrifugation contained significantly higher levels of miR‐146a‐5p than standard hucMSC‐EVs (Figure 3M). The time‐dependent uptake of PKH67‐labelled hucMSC‐EVs by NR8383 cells was observed at 2, 6 and 24 h. Specifically, at 24 h, the endocytosis rate of hucMSC‐EVs by NR8383 cells reached 96.6% (Figure 3N). To track the uptake of miR‐146a directly, cy5‐labelled miR‐146a was utilised to transfect hucMSCs, revealing cy5‐miR‐146a (red) in NR8383 cells 24 h after exposure to EVs‐miR‐146a (Figure 3O).

### 2.4 Engineered EVs‐miR‐146a exhibit superior inhibition of the NLRP3 inflammasome via IRAK1/TRAF6/NF‐κB pathway in the cold preservation and reperfusion NR8383 cell model compared with hucMSCs‐EVs treatment

**FIGURE 4:**
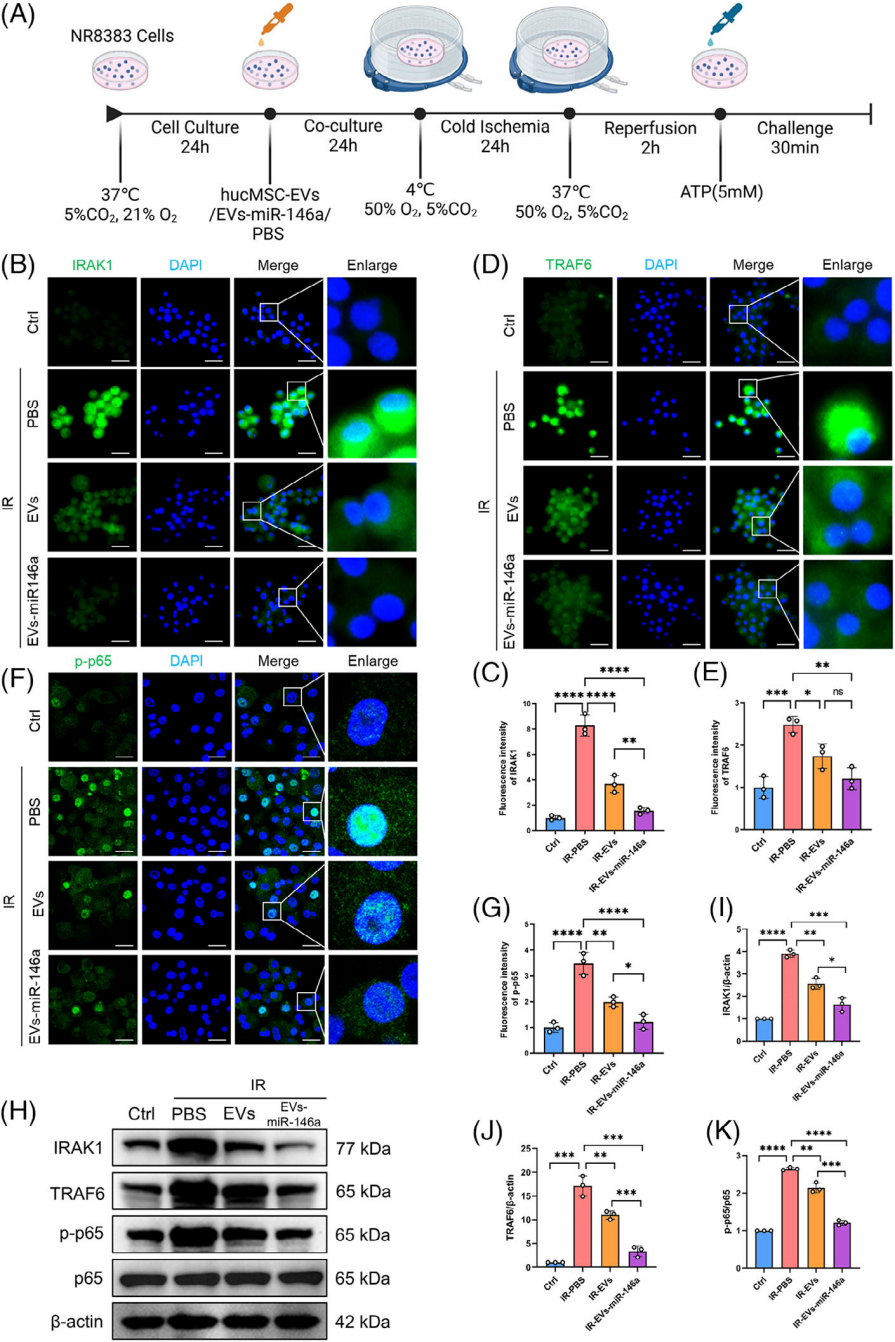
Engineered EVs‐miR‐146a inhibit the expression of target proteins IRAK1 and TRAF6 and suppress NF‐κB activation in vitro. These results further highlight the central role of IRAK1/TRAF6/NF‐κB signalling in initiating inflammatory responses following cold ischemia–reperfusion stress in alveolar macrophages. (A) Schematic workflow of the cold ischemia–reperfusion model in vitro. NR8383 cells were treated with hucMSC‐EVs, EVs‐miR‐146a or PBS for 24 h, followed by incubation at 4°C (50% O_2_, 5% CO_2_, balanced N_2_) for 24 h, 2 h of reperfusion at 37°C and stimulation with ATP (5 mM) for 30 min. (B–E) Immunofluorescence staining showing the expression and fluorescence intensity of IRAK1 (green) and TRAF6 (green) in NR8383 cells, with nuclei stained using DAPI (blue) (scale bar = 25 µm). IRAK1 and TRAF6 fluorescence intensity was highest in the IR‐PBS group, but was reduced following treatment with EVs and further reduced with EVs‐miR‐146a, with IRAK1 showing the greatest reduction after EVs‐miR‐146a treatment. (F and G) Immunofluorescence staining depicting the nuclear translocation and fluorescence intensity of phospho‐p65 (p‐p65) (green) in NR8383 cells (scale bar = 25 µm). p‐p65 nuclear translocation and fluorescence intensity were elevated in the IR‐PBS group, while treatment with EVs and EVs‐miR‐146a significantly reduced these levels, with a more pronounced effect observed in the IR‐EVs‐miR‐146a group. (H–K) Western blot analysis of IRAK1, TRAF6 and p‐p65 (Ser536) expression in NR8383 cells. Quantification of protein levels was performed using ImageJ, with normalisation to β‐actin. Data are presented as mean ± SD (*n* = 3). IR, ischemia reperfusion model based on NR8383 cell line; EVs, hucMSC‐EVs. All data are presented as mean ± SD. *p < .05, **p < .01, ***p < .001, ****p < .0001. Statistical comparisons between above groups were performed using unpaired Student's *t*‐tests.

In the rat LTx model, donor lungs underwent cold preservation and reperfusion, resulting in NLRP3 activation in AMs. To evaluate the impact of engineered hucMSC‐EVs on NLRP3 inflammasome activation in macrophages, we established a cold preservation and warm reperfusion cell culture model using NR8383 cells (Figure 4A). To simulate in vivo delivery, NR8383 cells were treated with hucMSC‐EVs, EVs‐miR‐146a or PBS for 24 h. After simulated cold preservation for 24 h and warm reperfusion for 2 h, treatment with both hucMSC‐EVs and EVs‐miR‐146a substantially diminished IRAK1 and TRAF6 expression levels. Notably, hucMSC‐EVs exhibited a more pronounced inhibitory effect compared with EVs‐miR‐146a. (Figure 4B–E,H–J). Furthermore, compared with the PBS‐pretreated group, the phosphorylation of p65 (p‐p65) was significantly reduced, and nuclear translocation was decreased in the hucMSC‐EVs and EVs‐miR‐146a groups. These findings were confirmed by immunofluorescence staining and Western blotting (Figure 4F,G,H,K). Likewise, the EVs‐miR‐146a group demonstrated a more significant effect than the hucMSC‐EVs group.

**FIGURE 5:**
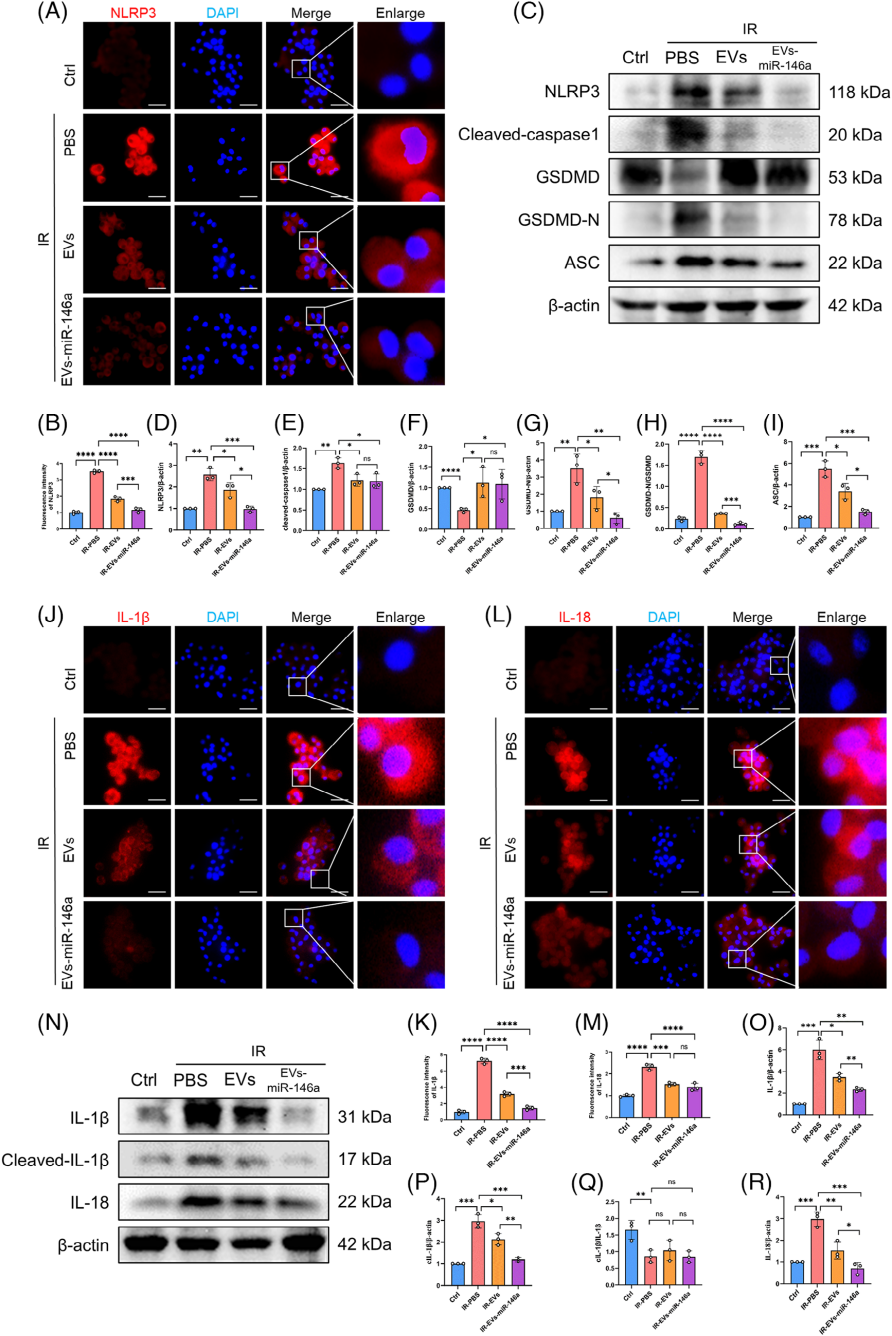
Engineered EVs‐miR‐146a attenuate NLRP3 inflammasome activation and inflammatory cytokines release in vitro. Consistent with Figure 4, inhibition of the IRAK1/TRAF6/NF‐κB pathway by engineered EVs‐miR‐146a effectively suppresses subsequent NLRP3 inflammasome activation. (A and B) Immunofluorescence staining showing the expression and fluorescence intensity of NLRP3 (red) in NR8383 cells, with nuclei stained using DAPI (blue) (scale bar = 25 µm). NLRP3 fluorescence intensity was elevated in the PBS‐treated group but significantly reduced following EVs and EVs‐miR‐146a treatment, with a more pronounced reduction in the EVs‐miR‐146a group. (C–I) Western blot analysis of NLRP3, cleaved caspase‐1, GSDMD, GSDMD‐N (active form) and ASC expression across different groups. Protein levels were quantified using ImageJ and normalised to β‐actin. Expression of NLRP3, cleaved caspase‐1, GSDMD, GSDMD‐N and ASC was elevated in the PBS‐treated group but reduced to varying extents following intervention with EVs and EVs‐miR‐146a. Data are presented as mean ± SD (*n* = 3). (J–M) Immunofluorescence staining showing the expression and fluorescence intensity of IL‐1β and IL‐18 (red) in NR8383 cells, with nuclei stained using DAPI (blue) (scale bar = 25 µm). The fluorescence intensity of IL‐1β and IL‐18 was elevated in the PBS‐treated group but reduced following treatment with EVs and EVs‐miR‐146a. (N–R) Western blot analysis of IL‐1β, cleaved IL‐1β and IL‐18 expression across different groups. Protein levels of IL‐1β, cleaved IL‐1β and IL‐18, as well as the ratio of cleaved IL‐1β to IL‐1β, were quantified using ImageJ and normalised to β‐actin. Elevated expression of IL‐1β, cleaved IL‐1β and IL‐18 in the PBS‐treated group was significantly reduced following intervention with EVs and EVs‐miR‐146a. IR, ischemia reperfusion model based on NR8383 cell line. EVs, hucMSC‐EVs. All data are presented as mean ± SD. *p < .05, **p < .01, ***p < .001, ****p < .0001. Statistical comparisons between groups were performed using unpaired Student's *t*‐tests.

The activation of NF‐κB serves as an initial signal for NLRP3 activation, thus regulating subsequent NLRP3 activation, while IRAK1 and TRAF6 enhance NF‐κB signalling, thereby playing critical roles in activating innate immune cells and triggering the subsequent inflammatory response. Moreover, NLRP3 inflammasome activation was inhibited in the groups treated with hucMSC‐EVs and EVs‐miR‐146a. Notably, the inhibitory effect was more prominent in the EVs‐miR‐146a‐treated group, as demonstrated by immunofluorescence staining and Western blot analysis (Figure 5A–D).

During NLRP3 activation, the inflammasome is formed through the recruitment of apoptosis‐associated speck‐like protein with a caspase recruitment domain (ASC) and pro‐caspase‐1. Western blot analysis showed that after NR8383 cells were subjected to simulated IR treatment, ASC expression was up‐regulated and cleaved caspase‐1 was activated (Figure 5C,E,I). The activated caspase‐1 then processes pro‐IL‐1β and pro‐IL‐18 into their mature, active forms, IL‐1β and IL‐18. Subsequently, Gasdermin D (GSDMD) is cleaved into GSDMD‐N (Figure 5C, F–H), inducing pyroptosis and leading to the secretion of IL‐1β and IL‐18. These elevated protein expressions were observed in the cold preservation and reperfusion NR8383 cell model, and administration of hucMSC‐EVs and EVs‐miR‐146a markedly inhibited NLRP3 activation and reduced the secretion of IL‐1β and IL‐18, with a more pronounced decrease observed following EVs‐miR‐146a intervention (Figure 5J–R).

### 2.5 Engineered EVs‐miR‐146a more effectively inhibited NLRP3 inflammasome activation through IRAK1/TRAF6/NF‐κB pathway and alleviated lung injury in a rat LTx model

**FIGURE 6:**
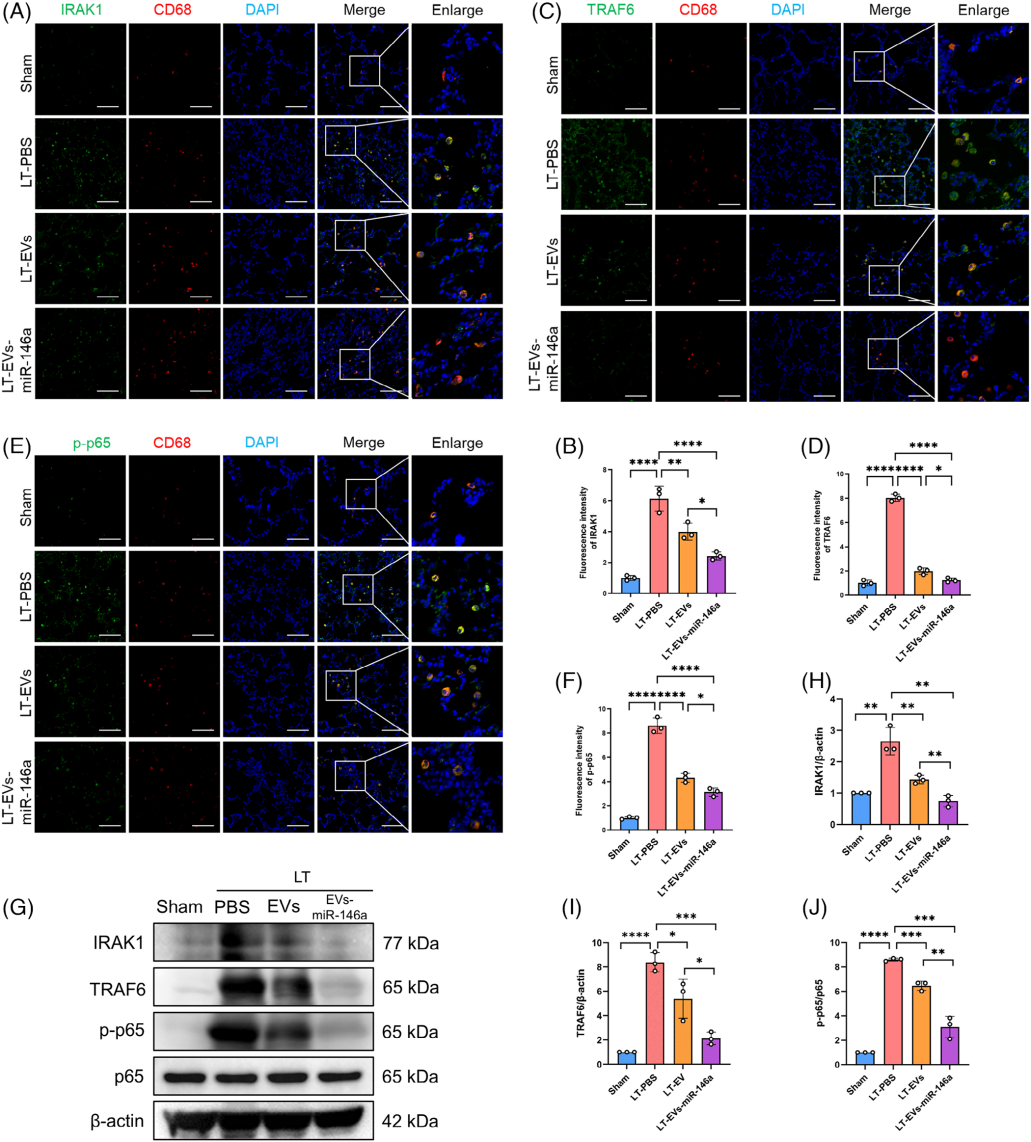
Engineered EVs‐miR‐146a more effectively inhibited NLRP3 inflammasome activation through IRAK1/TRAF6/NF‐κB pathway and alleviated lung injury in a rat LTx model. These results underscore the pivotal role of the IRAK1/TRAF6/NF‐κB signalling pathway in mediating the inflammatory response during lung IRI. (A–D) Representative images of double immunostaining for CD68 (macrophage marker, red) with IRAK1 (green) or TRAF6 (green) in lung tissues from the experimental groups. Nuclei are counterstained with DAPI (blue). Scale bar: 100 µm. Semi‐quantitative analysis of IRAK1 and TRAF6 immunofluorescence intensity was performed using ImageJ, showing an increased intensity in the LT‐PBS group, which is reduced following EVs and EVs‐miR‐146a treatment, particularly in the EVs‐miR‐146a group. (E and F) Representative images showing double immunostaining for CD68 (macrophage marker, red) and p‐p65 (green) across different experimental groups. The semi‐quantitative scores of p‐p65 immunofluorescence intensity were measured using ImageJ, highlighting reduced activation in the treatment groups. G‐J. Western blot analysis and quantification of IRAK1, TRAF6, p‐p65 and p65 expression levels in different experimental groups. Elevated expression of IRAK1 and TRAF6, as well as an increased p‐p65/p65 ratio, were observed in the LT‐PBS group, indicating NF‐κB activation. In contrast, expression levels and the p‐p65/p65 ratio were significantly reduced in the EVs and EVs‐miR‐146a groups, with the most pronounced reduction in the EVs‐miR‐146a group. Sham, sham surgical group of rats; LT‐PBS, lung transplantation group of rat tread with PBS; LT‐EVs, lung transplantation group of rat tread with hucMSC‐EVs; LT‐EVs‐miR‐146a, lung transplantation group of rat tread with engineered hucMSC‐EVs. All data are presented as mean ± SD. *p < .05, **p < .01, ***p < .001, ****p < .0001. Statistical comparisons between above groups were performed using unpaired Student's *t*‐tests.

In the rat LTx model, we further examined how hucMSC‐EVs and engineered EVs‐miR‐146a modulate NLRP3 inflammasome activation and lung injury. Immunofluorescence staining was performed to evaluate the expression levels of IRAK1, TRAF6 and phosphorylated p65 (p‐p65) in lung tissues, with AMs identified by CD68 (red) (Figure 6A–F). We observed significantly increased expression of IRAK1, TRAF6 and p‐p65 in CD68‐positive AMs, which was reduced in both the hucMSC‐EVs and EVs‐miR‐146a treatment groups, with EVs‐miR‐146a showing superior efficacy. Similar results were confirmed by Western blot analysis (Figure 6G–J).

**FIGURE 7:**
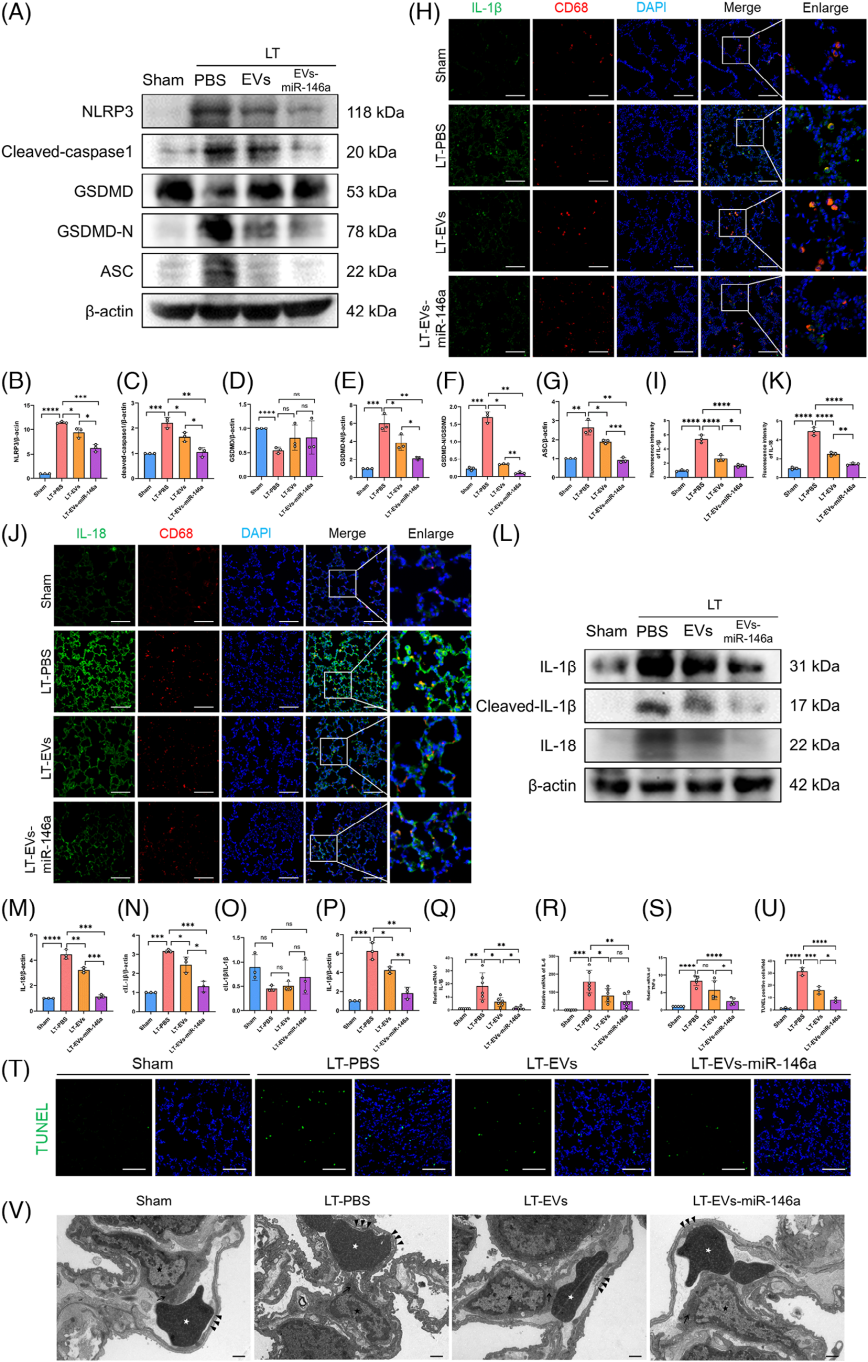
Engineered EVs‐miR‐146a more effectively inhibit NLRP3 inflammasome activation and alleviate lung injury in rat lung transplantation models. (A–G) Western blot analysis and semi‐quantification of NLRP3, cleaved‐Caspase 1, GSDMD, GSDMD‐N (active form) and ASC expression levels in each group. (H–K) Representative images of double immunostaining for CD68 (macrophage marker, red) with IL‐1β (green) and IL‐18 (green) in different experimental groups (scale bar: 25 µm). Semi‐quantitative analysis of IL‐1β and IL‐18 immunofluorescence intensity showed significantly increased expression in the LT‐PBS group, which was reduced to varying degrees following EVs and EVs‐miR‐146a interventions. Data are presented as mean ± SD (*n* = 3). (L–P) Western blot analysis and semi‐quantification of IL‐1β, cleaved IL‐1β (c‐IL‐1β, active form) and IL‐18 in the different groups. The expression levels of NLRP3, cleaved‐Caspase 1, GSDMD‐N/GSDMD, ASC, c‐IL‐1β/IL‐1β and IL‐18 were elevated in the LT‐PBS group but were reduced following EVs and EVs‐miR‐146a interventions. Data are presented as mean ± SD (*n* = 3). (Q–S) Relative mRNA expression levels of cytokines *IL‐1β, IL‐6* and *TNF‐α* in lung tissue were measured by qPCR. The LT‐PBS group showed increased expression of these inflammatory cytokines, while the EVs and EVs‐miR‐146a intervention groups exhibited decreased expression. Data are presented as mean ± SD (*n* = 6). (T and U) TUNEL staining of lung tissue sections to assess cell death (scale bar: 100 µm). Fluorescence intensity was quantified using ImageJ. Extensive cell death was observed in the LT‐PBS group, while EVs and EVs‐miR‐146a treatment attenuated cell death, with a more pronounced effect seen in the EVs‐miR‐146a group. Data are presented as mean ± SD (*n* = 3). (V) Transmission electron microscopy images of lung tissue sections from different groups (4000× magnification, scale bar: 1000 nm). The images show lung type II epithelial cells with specific osmophilic lamellar bodies (black star), red blood cells in the capillary (white star), the capillary basal membrane (black arrowheads) and specific osmophilic lamellar bodies (black arrows). The LT‐PBS group exhibited capillary endothelial barrier dissolution, rupture and basal membrane oedema (black arrowheads), whereas the EVs and EVs‐miR‐146a groups displayed reduced endothelial barrier disruption. Sham, sham surgical group of rats. LT‐PBS, lung transplantation group of rat tread with PBS. LT‐EVs, lung transplantation group of rat tread with hucMSC‐EVs. LT‐EVs‐miR‐146a, lung transplantation group of rat tread with engineered hucMSC‐EVs. All data are presented as mean ± SD. *p < .05, **p < .01, ***p < .001, ****p < .0001. Statistical comparisons between groups were performed using unpaired Student's *t*‐tests.

Following the activation of NF‐κB, we further examined NLRP3 inflammasome activation (Figure 7A–G). Western blotting demonstrated an up‐regulation of NLRP3, cleaved caspase‐1, GSDMD‐N/GSDMD and ASC in the LT‐PBS group. In contrast, treatment with hucMSC‐EVs and EVs‐miR‐146a markedly reduced the levels of these NLRP3‐related proteins in explanted lungs, with the engineered EVs‐miR‐146a exhibiting the most pronounced effect.

The expression levels of IL‐1β and IL‐18 were examined using Western blot analysis and immunofluorescence staining (Figure 7H–Q). The LT‐PBS group exhibited elevated levels of these cytokines, with co‐localisation indicating significantly higher expression in CD68‐positive AMs compared with other lung cells. Both hucMSC‐EVs and EVs‐miR‐146a interventions effectively suppressed IL‐1β and IL‐18 expression, with engineered EVs‐miR‐146a showing the best results. Additionally, we quantified the mRNA levels of inflammatory cytokines IL‐1β, IL‐6 and TNF‐α in lung tissue (Figure 7Q–S). Both hucMSC‐EVs and EVs‐miR‐146a interventions reduced the expression of these cytokines, though the effects were not uniform. Furthermore, both treatments effectively inhibited lung cell apoptosis. TUNEL staining of lung tissue showed that the number of apoptotic cells was markedly decreased after the application of hucMSC‐EVs and EVs‐miR‐146a (Figure 7T, U). Notably, the effect of EVs‐miR‐146a was more pronounced (Table 1).

**TABLE 1:** PCR primers.

Gene(Rat)	Sequence
IL‐1β	F:5′CTGAGCTCGCCAGTGAAATG3′
	R:5′TGTCCATGGCCACAACAACT3′
IL‐18	F:5′TGGCTGCTGAACCAGTAGAA3′
	R:5′ATAGAGGCCGATTTCCTTGG3′
TNF‐α	F:5′ACTGAACTTCGGGGTGATTG3′
	R:5′GTGGGTGAGGAGCAGGTAGT3′
GAPDH	F:5′AAGAAGGTGGTGAAGCAGGC3′
	R:5′TCCACCACCCAGTTGCTGTA3′

Transmission electron microscopy (TEM) of ultrathin sections of rat lung tissue was conducted to assess lung injury (Figures 7V). The LT‐PBS group exhibited oedema of the capillary basement membrane and discontinuity of the capillary endothelium, consistent with the lung injury scores obtained through H&E staining. This confirmed that the donor lungs experienced IRI following prolonged cold preservation. In contrast, lung tissue from the hucMSC‐EVs and EVs‐miR‐146a treatment groups showed more intact capillary structures and reduced oedema. Together with previous wet/dry ratio experiments, these findings indicate that the interventions alleviated lung oedema, with engineered EVs‐miR‐146a demonstrating the most effective results.

## 3 DISCUSSION

Effective donor lung preservation is essential for increasing utilisation and expanding the donor pool.^1^, ^2^, ^3^ This study is the first to employ nebulised miR‐146a engineered hucMSC‐EVs in LTx. Nebulisation enhances targeting of donor lungs, enabling direct delivery of therapeutic agents. By incorporating double‐stranded miR‐146a into EVs, we effectively reduced inflammatory responses by inhibiting NLRP3 inflammasome activation in AMs through targeting IRAK1 and TRAF6. These innovative findings indicate that nebulisation delivered hucMSC‐EVs engineered with miR‐146a can further enhance donor lung preservation and utilisation.

Donor lungs are subjected to ischaemia, cold preservation and reperfusion during the procurement‐to‐implantation period.^22^ Under these conditions, oxidative stress, metabolic dysregulation and cell death are triggered, alongside the release of damage‐associated molecular patterns. While hypothermia can reduce metabolic rates, various donor‐related changes can still provoke an inflammatory response.^6^, ^23^, ^24^ Our findings indicate that NLRP3 activation in AMs occurs in explanted lungs during prolonged cold ischemic time and reperfusion, resulting in the release of IL‐1β. Notably, treatment with hucMSC‐EVs alleviated IRI in our experimental rat LTx model.

Several studies,^25^, ^26^ including ours, have corroborated that MSC‐derived EVs can mitigate lung IRI through modulation of inflammatory pathways. The primary mechanism of action for MSC‐EVs is attributed to their miRNA content, which regulates biological processes by cleaving or inhibiting target gene translation.^12^, ^27^ Our study specifically discovered an increase in miR‐146a‐5p levels in in hucMSC‐EVs, which has demonstrated anti‐inflammatory properties in various disease models. Similar to previous reports,^18^, ^28^ we predicted that miR‐146a‐5p targets IRAK1 and TRAF6, key effector proteins in the TLR4 signalling pathway, which, when recruited to MyD88, activate downstream NF‐κB signalling.^29^

Activation of the NLRP3 inflammasome requires two signals.^30^ The initial signal activates the NF‐κB pathway, thereby up‐regulating essential genes – including NLRP3, pro‐IL‐1β and pro‐IL‐18 – that are critical for inflammasome assembly. Thus, our approach to suppressing NLRP3 activation via inhibition of NF‐κB signalling is both feasible and effective. However, the therapeutic efficacy of MSC‐derived EVs may vary due to differences in cell culture conditions or the extracellular microenvironment. To enhance their therapeutic potential, various strategies, including pre‐conditioning of MSCs and modifications of cell surface properties, have been employed. These approaches can yield engineered EVs with superior targeting capabilities and stability compared with native EVs^31^, ^32^

Exogenous miR‐146a‐5p has been proven to trigger a strong immunostimulatory response through TLR7 activation via a UU‐containing motif.^33^ Thus, directly introducing miR‐146a‐5p mimics into hucMSC‐EVs may not be the most effective strategy. In contrast, the duplex precursor miR‐146a does not trigger an immune response. When miR‐146a binds to Argonaute proteins, it forms the RNA‐induced silencing complex (RISC), which then targets the 3′ UTR of IRAK1, thereby dampening the host immune response. Consequently, we selected miR‐146a as a candidate miRNA. The targeted delivery of miRNAs into EVs through parental cell engineering is an efficient method for generating engineered EVs. In our study, we constructed engineered EVs overexpressing miR‐146a (EVs‐miR‐146a) by transfecting MSCs with a miR‐146a mimic. Treatment with EVs‐miR‐146a resulted in reduced IRAK1 protein expression both in vivo and in vitro, ultimately suppressing NLRP3 activation and mitigating IRI in LTx.

Our study employed inhalation of MSC‐EVs in donor lungs to attenuate IRI following experimental LTx in rats. Our rat LTx model achieved a success rate above 90%, demonstrating the reliability and consistency of our experimental system. This high success rate validates our methodology and provided a solid foundation for evaluating the therapeutic efficacy of engineered EVs. As a non‐invasive strategy, nebulisation therapy represents an effective drug delivery method for lung diseases. Despite limited research on nebulised MSC‐EVs in the context of LTx, some studies have suggested their potential. For instance, one investigation^34^ found that inhaled MSC‐EVs reduced inflammation in an LPS‐induced acute lung injury model more effectively than tail vein injections. Another study^35^ indicated superior distribution of mRNA and protein cargo in the lungs following nebulisation of lung‐derived EVs compared with lipid nanoparticles. These findings, alongside our own, suggest that nebulised MSC‐EVs may represent a feasible and effective therapeutic strategy for pulmonary drug delivery.

Management of donor lungs poses significant challenges due to physiological changes following brain death. Current treatment methods, including lung‐protective ventilation strategies and bronchoscopy, have primarily focused on systemic medications such as diuretics and hormonal therapies, which can affect multiple organs within the same donor.^3^ In contrast, nebulised administration provides a localised treatment option for donor lungs. Nebulised MSC‐EVs predominantly target bronchiolar and parenchymal cells, as the blood–gas barrier restricts their entry into the circulatory system.^34^, ^35^ Recent advances, such as normothermic ex vivo lung perfusion (EVLP), have gained importance in assessing and repairing injured donor lungs.^36^ Some studies have reported positive outcomes when using MSCs and MSC‐EVs in conjunction with EVLP; however, there remains a scarcity of effective therapies utilising engineered EVs in this context, indicating a need for further research.^37^, ^38^

Importantly, while previous studies have concentrated on donor lung management prior to organ retrieval in rat models, the efficacy and applicability of such treatments warrant validation through prospective clinical trials. In this study, we isolated hucMSC‐EVs using differential ultracentrifugation, which is regarded as the gold standard for exosome isolation due to its ability to achieve higher purity than alternative methods, such as polyethylene glycol‐induced precipitation.^39^ Nonetheless, challenges remain in avoiding contamination with proteins and lipoproteins, and the reliance on specialised equipment and trained personnel may limit the application of this technique in some medical centres. Despite these obstacles, advancements in isolation technologies, including tangential flow filtration and size‐exclusion chromatography, offer promising solutions for improving the yield and quality of EVs for clinical applications. Future studies will leverage these new methodologies to enhance the clinical utility of EVs in LTx.

## 4 MATERIALS AND METHODS

### 4.1 Animals

Lewis rats were procured from Changzhou Kavins Experimental Animal Co. Ltd. The animals were maintained under specific pathogen‐free conditions at the Animal Experimental Center of Wuxi People's Hospital, with free access to food and water. Ambient temperature was maintained at 22°C with a 12‐h light/dark cycle. Adult male rats weighing 300–350 g were used in this study.

### 4.2 Preparation and identification of MSCs

MSCs were derived from the Wharton's jelly of human umbilical cords using an explant culture technique. The cells were cultured in DMEM/F12 medium containing 10% FBS and penicillin/streptomycin (100 U/100 mg/mL; Thermo Fisher Scientific, Burlington, Canada) at 37°C in a 5% CO₂ environment. Their identity was confirmed by flow cytometry, which demonstrated the expression of positive markers (CD73, CD105 and CD90) and the absence of negative markers (CD34, CD45, CD79α, CD11b and HLA‐DR). The trilineage differentiation potential of MSCs was induced using appropriate differentiation media. Cells were cultured in adipogenic differentiation medium (PD‐019; Procell, Wuhan, China) and osteogenic differentiation medium (PD‐017; Procell) for 3 weeks, and in chondrogenic differentiation medium (PD‐018; Procell) for 4 weeks.

### 4.3 Isolation and characterisation of hucMSC‐EVs

HucMSC‐derived EVs were isolated through differential ultracentrifugation as previously described.^39^ HucMSCs at passages 5–6 were cultured in medium containing exosome‐free FBS for 48 h. The conditioned medium was collected and sequentially centrifuged at 300×*g* for 10 min and 3000×*g* for 30 min to remove cells and debris. Following a 10 000×*g* centrifugation for 40 min to eliminate apoptotic bodies and larger vesicles, the supernatant was passed through a 0.22 µm filter. Subsequently, ultracentrifugation was conducted at 120 000×*g* for 90 min, after which the supernatant was discarded, and the pellet containing EVs was resuspended in DPBS. This ultracentrifugation step was repeated, yielding a gelatinous precipitate containing purified EVs, all conducted at 4°C. TEM was utilised to examine EV morphology, while NTA quantified particle concentration and size distribution. Specific exosomal markers (positive markers: TSG101, CD9 and CD63; negative marker: calnexin) were examined via Western blotting. The isolated hucMSC‐EVs were subsequently utilised for both in vivo and in vitro experiments.

### 4.4 LTx model in rats

Twenty‐four hours before harvesting the lungs from the donor rats, the donor rats were administered nebulised hucMSC‐EVs or PBS using a vibrating mesh nebuliser (Contec Ltd., Qinhuangdao, China). The hucMSC‐EVs were prepared at a relative concentration of 1 mg dissolved in 1 mL of PBS. The nebulisation duration was 5 min, and it was performed only once. For a picture of the nebulised donor rats, please refer to Figure S1. Donor rats were anaesthetised with 5% isoflurane and positioned supine. Following hair removal and disinfection, a median thoracoabdominal incision was performed. Heparin (500 IU/kg) was administered via the inferior vena cava, and the pulmonary artery was perfused with a cold low potassium dextran (LPD) solution (4°C, 20 mL). The heart‐lung blocks were then excised, inflated with a 50% oxygen and 50% nitrogen gas mixture (2.5 mL), and the main bronchus was ligated. The heart‐lung block was stored on ice in cold LPD solution at 4°C for 12 h. An orthotopic left LTx model was established using the cuff technique. After 2 h of reperfusion, arterial blood was collected from the left pulmonary vein for blood gas analysis (i‐stat300G; Abbott Pharmaceutical Co. Ltd., Lake Bluff, IL, USA). At the conclusion of each experiment, grafted lungs were harvested and perfused with 0.9% saline. For the assessment of lung wet/dry ratios, the wet weight of each lung tissue sample was normalised to 0.1 g, with dry weight measured after heating the tissue at 65°C for 48 h. Lung injury scores were calculated in accordance with American Thoracic Society guidelines.

### 4.5 Simulated ischaemia/reperfusion cell culture model

The NR8383 rat AM cell line (Procell) was employed to develop an ischaemia/reperfusion cell culture model. NR8383 cells were seeded in six‐well plates and cultured in low‐serum Ham's F‐12K (Kaighn's) medium (5% FBS). Cells were treated with hucMSC‐EVs, EVs‐miR‐146a or PBS for 24 h to simulate their in vivo delivery for donor management. After treatment, NR8383 cells were incubated in a hypoxic chamber at 4°C with 50% O_2_, 5% CO_2_ and the balance of N_2_ for 24 h. Following 2 h of reperfusion at 37°C, the cells were moved to a standard culture incubator at 37°C and treated with ATP (5 mM) for 30 min.

### 4.6 Human scRNA‐seq

Twelve human lung biopsies were collected at the end of the cold ischemic period and after 2 h of reperfusion for single‐cell sequencing. Details regarding sample collection, data preprocessing and quality control are outlined in our previous study.^19^ The GSE code of sequence data was GSE220797. The ‘FeaturePlot’ and ‘DotPlot’ were performed based on R package ‘Seurat’.

### 4.7 Construction of miR‐146a engineered hucMSC‐derived EVs

MiR‐146a engineered hucMSC EVs were constructed using a parental cell engineering approach.^40^ HucMSCs at passages 5–6 were transfected with 60 nM of miR‐146a mimic (Ribobio, Guangzhou, China) following the manufacturer's protocol. After 24 h, the cells were washed and cultured in exosome‐free FBS medium. The culture supernatant was collected after an additional 48 h of incubation, and engineered EVs were isolated using differential ultracentrifugation as previously described.

### 4.8 Uptake of exosomes by lung tissues and NR8383 cells

HucMSC‐EVs were labelled with PKH67 (Sigma) following the manufacturer's instructions. The rats were nebulised with PKH67‐labelled EVs, as previously described. The inflated lungs were harvested and rapidly immersed in liquid nitrogen after 24 h. Frozen lung sections were prepared for immunofluorescent analysis. NR8383 cells were fixed using 4% PFA after incubated with PKH67‐labelled exosomes at separate time points (2, 6, 24 h). Immunofluorescence analysis of these cells was performed as previously described.

### 4.9 Immunohistochemistry and immunofluorescence staining

According to a previously reported method,^34^, ^35^ multicolour fluorescence staining was used to determine the co‐localisation of the target proteins and AM (CD68) in lung tissue. In the different intervention groups, we assessed IRAK1, TRAF6, p‐p65, NLRP3, IL‐1β and IL‐18, with DAPI staining for cell nuclei. In addition, cell immunofluorescence staining was conducted to evaluate the expression of IRAK1, TRAF6, NLRP3, IL‐1β and IL‐18, along with the nuclear translocation of p‐p65, after the cell model was established. Briefly, NR8383 cells were seeded in glass‐bottom dishes. After treatment, cells were stained according to the standard protocol. DAPI was used to stain the cell nuclei, and imaging was conducted using a confocal microscope (Leica TCS SP8).

### 4.10 Ethical approval

This study was approved by the ethics committees of Zhejiang University, Wuxi People's Hospital(No. 2023‐01) and Wuxi Second People's Hospital (No. 2022‐Y‐119). All experiments were performed in compliance with the applicable ethical guidelines and regulations.

### 4.11 Statistics

All experiments were conducted independently at least three times. Data analysis was carried out using GraphPad Prism 10.1.2 software (GraphPad Software Inc., La Jolla, CA, USA). Results are expressed as mean ± standard deviation (SD). Comparisons between two groups were performed using unpaired *t*‐tests, with statistical significance set at *p* < .05.

## AUTHOR CONTRIBUTIONS

*Writing—original draft, conceptualisation, visualisation and data curation*: Xiucheng Yang. *Methodology and validation*: Shanchao Hong. *Software and formal analysis*: Tao Yan. *Methodology and validation*: Mingzhao Liu. *Writing—review and editing and conceptualisation*: Mingyao Liu. *Data curation*: Jin Zhao. *Resources*: Bingqing Yu. *Software*: Di Wu. *Resources*: Jingbo Shao. *Conceptualisation and supervision*: Man Huang. *Conceptualisation, writing—review and editing, funding acquisition and project administration*: Jingyu Chen.

## CONFLICT OF INTEREST STATEMENT

The authors declare no conflicts of interest.

## Supporting information



Supporting Information

## Data Availability

The analysed data sets generated during the present study are available from the corresponding author on reasonable request.
