# Supplementary material for: MiR‐146a engineered extracellular vesicles derived from mesenchymal stromal cells more potently attenuate ischaemia–reperfusion injury in lung transplantation
Source: Clin Transl Med. 2025 Apr 7;15(4):e70298. doi: 10.1002/ctm2.70298 (PMC11975614; doi:10.1002/ctm2.70298)
Supplement: Supplementary file 1 — Supporting Information [file CTM2-15-e70298-s001.docx]

MiR-146a Engineered Extracellular Vesicles derived from Mesenchymal Stromal Cells More Potently Attenuate Ischemia-Reperfusion Injury in Lung Transplantation

**Supplementary Materials**

**Figure S1 Nebulization of hucMSC-EVs in Donor Rats**


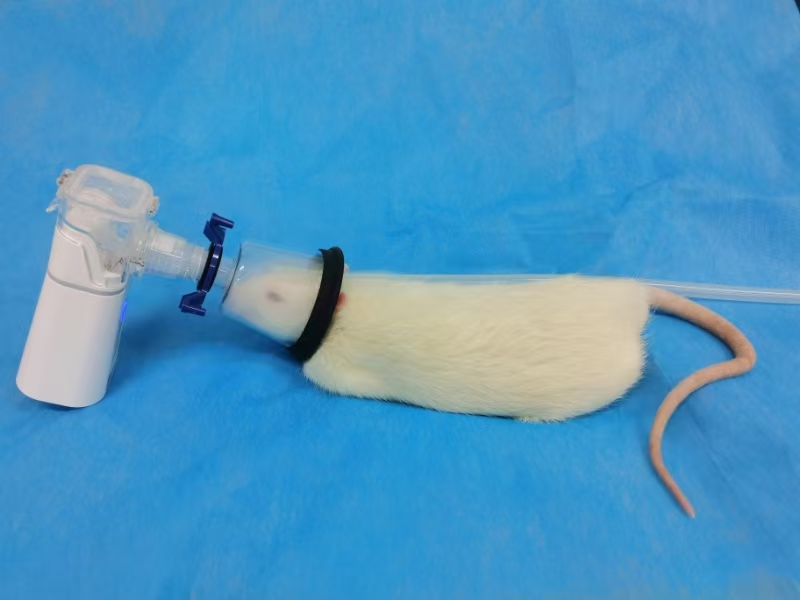


**Figure S1 legend**

Donor rats were treated with nebulized hucMSC-EVs or PBS using a vibrating mesh nebulizer 24 hours before lung procurement. The nebulizer was connected to an anesthetic mask for the administration of the treatment.

**Figure S2 Characterization of hucMSCs**


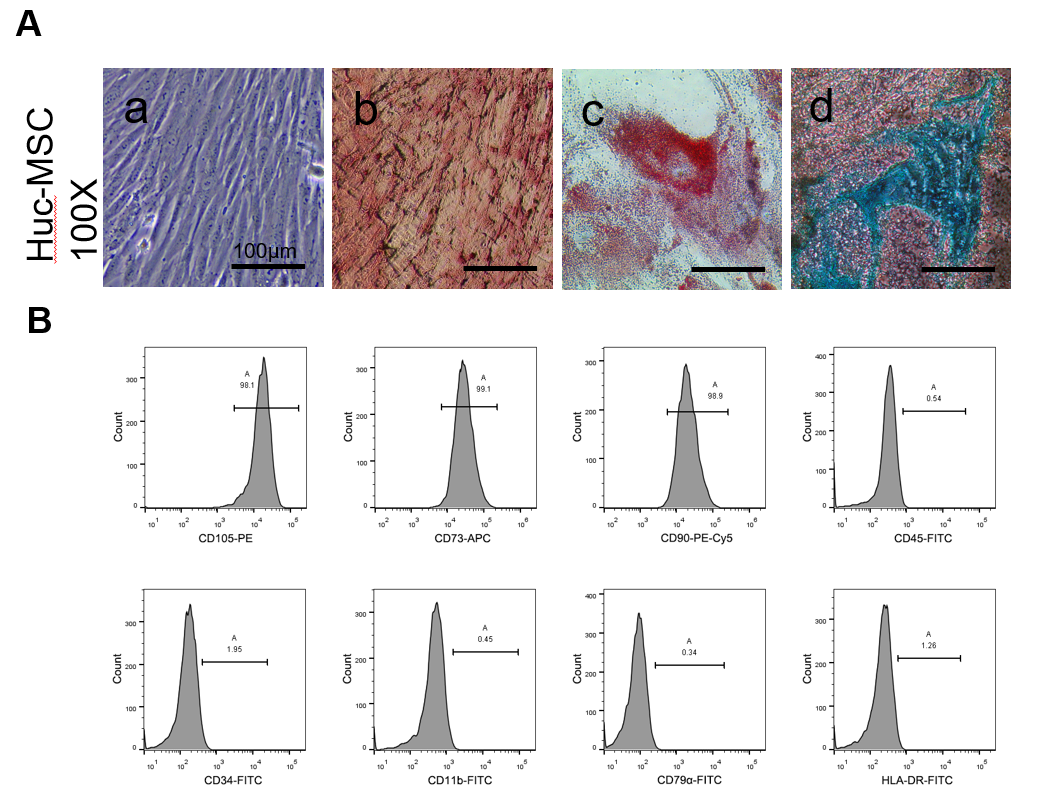


**Figure S2 legend**

A. The cultured hucMSCs were uniformly long spindle-shaped cells that have the potential to differentiate into osteoblasts, adipocytes, and chondroblasts.

B. Flow cytometry analysis of cells was positive for the expression of stem cell markers such as CD105, CD73, and CD90, and negative for the expression of markers such as CD45, CD34, CD11b, CD79α, and HLA-DR.

**Figure S3 Pulmonary Edema and Consolidation in Rat Orthotopic Left Lung Transplantation Model**


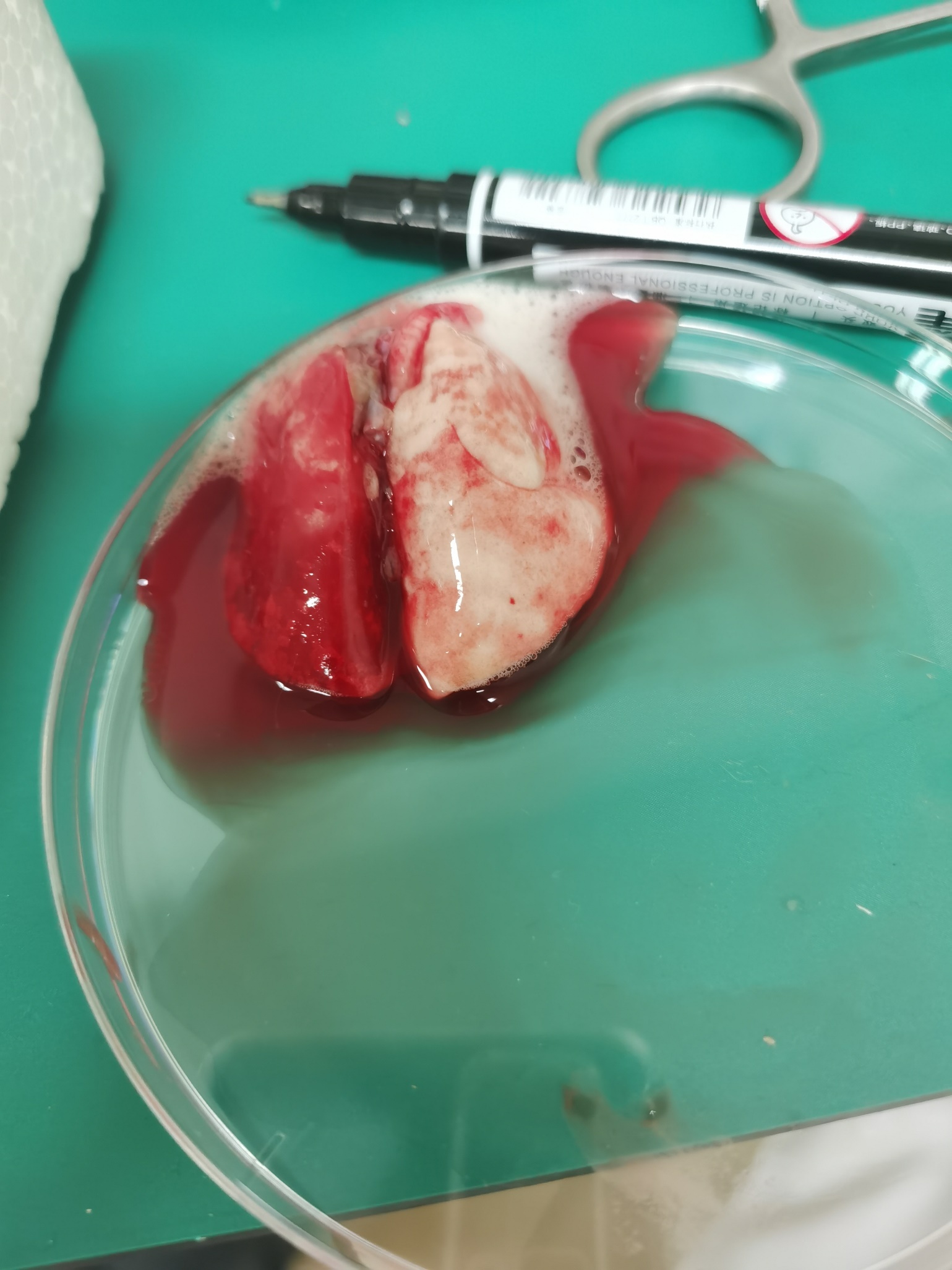


**Figure S3 legend**Two hours after orthotopic left lung transplantation in rats, the transplanted lung exhibited severe edema and consolidation. Upon dissection of the trachea, a significant amount of pulmonary edema fluid was observed to emerge from the trachea.

**Figure S4 NLRP3 Expression and Localization in Alveolar Macrophages**


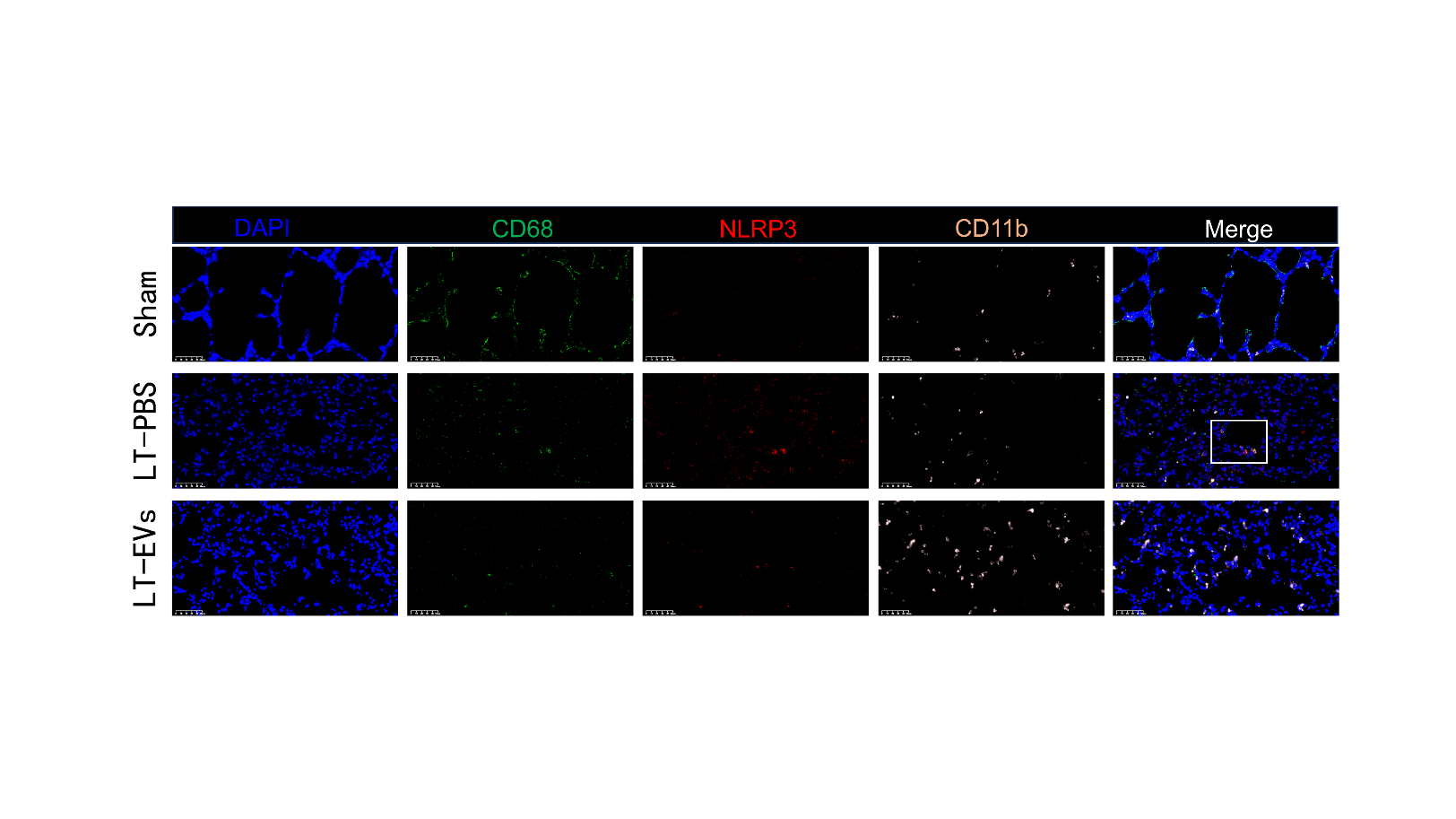


**Figure S4 legend**Immunofluorescence staining was used to assess NLRP3 expression (red) in lung tissue. NLRP3 was primarily localized in CD68-positive, CD11b-negative alveolar macrophages (White rectangle). Higher expression levels of NLRP3 were observed in the PBS-treated group compared to the hucMSC-EVs-treated group.

**Figure S5 Measurement of the PaO2/FiO2 from blood collected from the left pulmonary vein.**


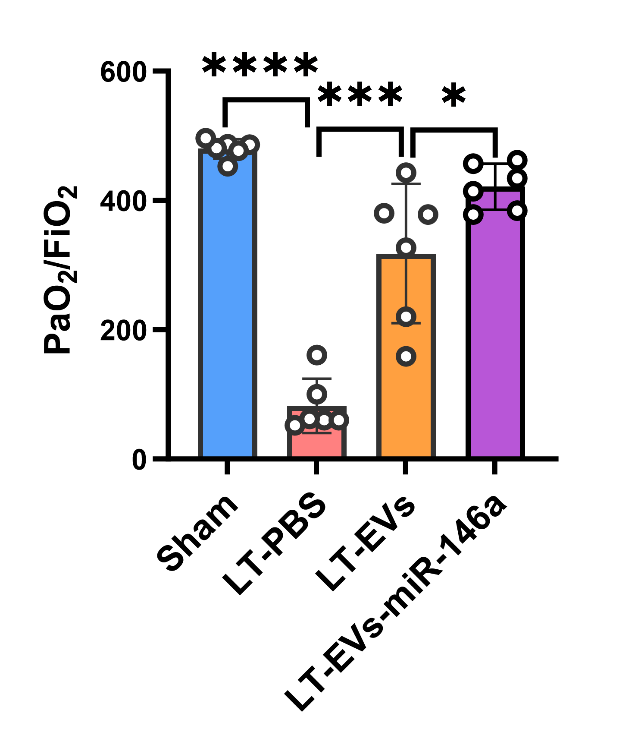


**Figure S5 legend**

Measurement of the PaO2/FiO2 from blood collected from the left pulmonary vein. Compared to the LT-EVs group, LT-EVs-miR-146a treatment significantly increasing the pulmonary venous PaO2/FiO2 (n=6).

Sham, sham surgical group of rats. LT-PBS, lung transplantation group of rats tread with PBS. LT-EVs, lung transplantation group of rats tread with hucMSC-EVs. All data are presented as mean ± SD. *p < 0.05, **p < 0.01, ***p < 0.001, ****p < 0.0001. Statistical comparisons between groups were performed using unpaired Student's t-tests.

**Supplementary Materials and Methods**

**1.1 miRNA sequencing**

Total RNA was extracted from three independent hucMSC-exo samples using the miRNA Isolation Kit (DP503; Tiangen Biotech, Beijing, China) following the manufacturer’s protocol. RNA concentration was measured using a NanoDrop and analyzed with an Agilent 2100 bioanalyzer (Thermo Fisher Scientific, MA, USA). A miRNA library was constructed using 1 μg of total RNA, and the final ligation PCR products were sequenced on the BGISEQ-500 platform (BGI, Shenzhen, China).

**1.2 Dual‑luciferase reporter assay**

The 3’ UTR sequence of IRAK1, containing either the wild-type (WT) or mutant (Mut) predicted miR-146a-5p binding sites, was cloned into the pmirGLO dual-luciferase vector. HEK-293T cells were co-transfected with the luciferase reporter plasmid and either NC-mimic or miR-146a-5p mimic. After 48 hours, luciferase activity was measured, with firefly luciferase activity normalized to Renilla luciferase activity.

**1.3 qRT‑PCR**

Total RNA was extracted from lung tissues and cell samples using the RNAeasy™ Animal RNA Isolation Kit with a Spin Column (Beyotime, Shanghai, China), following the manufacturer’s instructions. Reverse transcription was performed using the HiScript III 1st Strand cDNA Synthesis Kit with gDNA wiper (Vazyme, Nanjing, China). qPCR was carried out with the ChamQ Universal SYBR qPCR Master Mix (Vazyme, Nanjing, China) and analyzed using an ABI 7500 Fast System (Thermo Fisher Scientific). mRNA and miRNA expression levels were normalized to GAPDH and U6 reference genes, respectively. Cel-miR-39 was used as a spiked-in control to normalize miRNA quantification in hucMSC-exo. Data were calculated using the 2-ΔΔCt method. The sequences of the primers used in this study are provided in Table 1, with all primers designed and synthesized by RiboBio (Guangzhou, China).

**1.4 Western blot analysis**

Proteins were extracted from lung tissue and cells using RIPA buffer (Beyotime, Shanghai, China), supplemented with phosphatase and protease inhibitors. Western blot analysis was performed by separating proteins via SDS–polyacrylamide gel electrophoresis (SDS–PAGE, Beyotime, Shanghai, China), followed by electroblotting onto activated polyvinylidene difluoride (PVDF) membranes using Western blot Rapid Transfer Buffer (Epizyme, Shanghai, China), according to standard protocols. To determine the expression of target proteins, specific antibodies were used, including CALNEXIN (1:1000 dilution; Abcam; ab133615), CD63 (1:1000 dilution; Abcam; ab134045), TSG101, (1:1000 dilution; Abcam; ab125011), CD9 (1:1000 dilution; Abcam; ab263019), β-actin (1:1000 dilution; Proteintech; 66009-1-Ig), CD6 (1:1000 dilution; Abcam; ab283654), NLRP3 (1:1000 dilution; Abcam; ab270449), IRAK1 (1:1000 dilution; Proteintech; 10478-2-AP), TRAF6 (1:1000 dilution; Abcam; ab40675), NF-κB p65 (1:1000 dilution; Proteintech; 10745-1-AP), phospho-NF-κB p65 (1:1000 dilution; CST; 3033), ASC (1:1000 dilution; Abcam; ab309497), Cleaved Caspase 1 (1:1000 dilution; CST; 89332), GSDMD (1:1000 dilution; Abcam; ab219800), GSDMD-N (1:1000 dilution; CST; 10137), IL-1β (1:1000 dilution; Abcam; ab283818), Cleaved IL-1β (1:1000 dilution; CST; 63124), IL-18 (1:1000 dilution; Proteintech; 10663-1-AP). The image grayscale values were analyzed using ImageJ 1.51 software (NIH, Bethesda, MD, USA), and the relative protein expression levels were calculated accordingly.

**1.5 The detection of inflammatory factors by ELISA**

Lung tissue was homogenized, centrifuged, and the supernatant was collected for ELISA analysis. Similarly, cell supernatants were also collected for ELISA analysis. Rat cytokine ELISA kits for IL-1β, TNF-α, and IL-6 were obtained from Lianke Biological Co., Ltd., and assays were conducted following the manufacturer’s guidelines.

**1.6 Histological study**

Rat lungs from each group were harvested to assess cell infiltration and alveolar collapse in LTx. Tissue sections were deparaffinized, stained with hematoxylin and eosin (HE), and examined under light microscopy (Leica, Germany). The images were analyzed using a numerical scale, and Image-Pro Plus software was employed to quantify the lesion area in the pathological sections. Lung injury scores were calculated following the American Thoracic Society guidelines.
